# Standardized Ileal Digestibility of Amino Acids in L‐Amino Acids Sources and Single Cell Protein Biomass Coproduct for Pigs

**DOI:** 10.1111/asj.70177

**Published:** 2026-04-04

**Authors:** Stephane Alverina Briguente da Motta, Nathana Rudio Furlani, Antônio Carlos Macedo Lourenço, Pedro Silva Carelli, Lucimauro da Fonseca, Bruno Teixeira Ramos, Gabriel Ribeiro Braga, Melissa Izabel Hannas

**Affiliations:** ^1^ Department of Animal Science Universidade Federal de Viçosa Viçosa Minas Gerais Brazil

**Keywords:** amino acids, digestibility, L‐tryptophan, pigs, single‐cell protein biomass

## Abstract

Nine male pigs, which average 44.8 ± 3.6 kg body weight, were surgically fitted with a T cannula in the terminal ileum of the small intestine. The animals were assigned to a triplicated 3 × 2 incomplete Latin square design, consisting of three treatments: a complete mixture of industrial amino acids (L‐AAs), a single cell protein (SCP) biomass coproduct, and an nitrogen‐free diet. The animals were fed twice a day, with 5 days of adaptation to the experimental diets, followed by 2 days of continuous collection of ileal digesta for 10 h. Titanium dioxide was included in all diets at 0.5% as an indigestibility marker, and the N‐free diet was used to estimate basal endogenous ileal losses of amino acids (AA). The standardized ileal digestibility (SID) of crude protein was higher (*p* < 0.05) for L‐AAs compared to SCP (101.3% vs. 73.4%). For the indispensable and dispensable AA, the L‐AAs also presented higher SID (*p* < 0.05) to SCP. Specifically for L‐tryptophan, the SID of SCP was 89.2%. These results highlight that L‐AAs are highly digestible, with SID values close to 100%, whereas SCP demonstrates viability as a source of indispensable and dispensable AA in swine nutrition.

## 1 Introduction

Advancements in genetic selection and the increasing nutrient demands required to sustain rapid growth in modern pigs have intensified the need for industrial amino acid (AA) supplementation as an alternative to conventional protein sources, such as soybean meal, in pig diets. Over time, the growing commercial availability of industrial AA has enabled reductions in dietary crude protein (CP) levels, promoting substantial environmental and economic benefits through improved nutrient utilization and decreased nitrogen (N) excretion (Miranda et al. 2015).

Concurrently, there has been a growing interest in incorporating industrial coproducts as alternative protein sources. These coproducts offer a cost‐effective and sustainable approach to feed formulation, reinforcing the importance of their integration into pig diets to optimize both production efficiency and environmental sustainability.

Within this context, residual biomass generated during the industrial production of AA, such as L‐lysine, L‐threonine, and L‐tryptophan, can be utilized as sources of AA. These AAs are obtained through bacterial fermentation using mutant strains of *Corynebacterium glutamicum* , in a culture medium enriched with carbohydrates and other nutrients (Leuchtenberger et al. 2005). After fermentation, the target AA is purified and extracted from the biomass (Hermann 2003; Wensley et al. 2020), leaving behind a coproduct rich in crude protein and containing a balanced profile of indispensable and dispensable AA (Almeida et al. 2014). This residual biomass, also known as single‐cell protein (SCP), represents a potentially valuable ingredient for swine nutrition.

However, to effectively incorporate these fermentation‐derived coproducts into swine diets, a precise characterization of their nutritional value is required. Determining the standardized ileal digestibility (SID) of AA provides a reliable estimate of bioavailability, thereby supporting more accurate and efficient diet formulation.

Despite the recognized importance of industrial AA, their SID values require more precise definition. Most published data have been generated using diets that include conventional protein sources, which may overestimate digestibility due to background nitrogen and endogenous amino acid contributions. Furthermore, the digestion and absorption kinetics of free amino acids differ from those of amino acids bound within complex protein matrices, influencing intestinal physiology and overall nutrient utilization (Eugenio et al. 2023). Therefore, establishing accurate SID values for both purified industrial amino acids and fermentation‐derived coproducts is essential to improve diet formulation, optimize nutrient efficiency, and support a more sustainable swine production system.

In this context, the objective of this study was to determine the standardized ileal digestibility of a mixture of industrial amino acids (L‐AAs) in a chemical defined AA diet and single cell protein (SCP) biomass coproduct for growing pigs.

## 2 Materials and Methods

The protocol for this experiment was reviewed and approved by the Ethics Committee for the Use of Production Animals of the Federal University of Viçosa (Protocol Number 59/2023) and followed the rules of the National Council for the Control of Experimental Animals. This research was carried out at the Teaching, Research and Extension Unit in Swine Production and Nutrition of the Department of Animal Science of the Federal University of Viçosa, Viçosa, Minas Gerais, Brazil.

### 2.1 Animal Surgery

Prior to the experiment, nine growing male pigs (70 days of age and 30.0 kg BW) were surgically fitted with a simple T cannula in the distal ileum, 20 cm from the ileocecal valve, using a technique adapted from Donkoh et al. (1994). The animals were kept fasting for 12 h before the procedure to minimize contamination with digesta during the operation. Subsequently, the animals were anesthetized under general anesthesia, induced using a combination of ketamine (20 mg per kg BW) and xylazine (2 mg/kg BW), with tiletamine–zolazepam (2 mg/kg of BW) administered as needed to maintain an adequate anesthetic plane throughout the procedure. Local anesthesia was provided at the surgical site using lidocaine (4 mg/kg of BW).

The animals were then placed in the left lateral decubitus position and extensively scraped to ensure asepsis at the surgical site. A vertical incision of 5–6 cm was made in the flank region, positioned 3–4 cm from the caudal vertebra to the last rib. The small intestine was then exposed and manipulated. A 2‐ to 2.5‐cm incision was made along the antimesenteric side of the small intestine, 10–15 cm from the cranial segment to the ileocecal junction. A suture was placed around the incision, and a cannula was inserted and fixed to the intestine. A 1‐cm diameter incision in the skin was made 3–4 cm dorsally to the initial incision. Subsequently, all tissues were sutured.

Pigs were housed in individual pens with 2.3 m × 2.16 m × 0.95 m (length × width × height), equipped with individual dry feeder and bowl‐type drinker. The shed was previously sanitized with soap and water and later disinfected with quicklime, water, and disinfectant solution. For 5 days after surgery, pigs received therapeutic levels of broad‐spectrum antibiotics as a prophylactic measure, and postoperative pain and inflammation were controlled using a nonsteroidal anti‐inflammatory drug.

During the postoperative period, the animals received feed gradually on the first day and, later, ad libitum. The diet was formulated based on corn and soybean meals to meet all nutritional requirements according to the Brazilian Tables for Poultry and Swine (BTPS) by Rostagno et al. (2017). The skin suture was removed 15 days after surgery. The ileal digestibility test with a simple T cannula was performed 15 days after surgery, considering the recovery of all animals.

### 2.2 Diets and Experimental Design

Nine growing male pigs (Agroceres PIC, Camborough × AGPIC 412) with initial body weight of 44.7 ± 3.6 kg were distributed in a triplicated 3 × 2 incomplete Latin square design, with three diets and two periods of 7 days in each square, totaling six observations per treatment and 18 total observations. In the second period, the animals were changed treatment to exclude the effects of the individual on digestibility values. The experimental treatments consisted of L‐AAs, a SCP, and an N‐free diet. The mixture of L‐AAs evaluated included L‐lysine, L‐methionine, L‐tryptophan, L‐arginine, L‐histidine, L‐isoleucine, and L‐valine (CJ CheilJedang; Brazil and Korea); L‐glycine and L‐leucine (Zhangjiagang Specom Biochemical Co. Ltd., China); L‐phenylalanine (Shijiazhuang Shixing Amino Acid Co. Ltd., China); L‐threonine (Evonik Industries); and L‐glutamic acid (Ajinomoto Co. Inc.). The SCP consisted of a fermented microbial biomass derived from the residual substrate remaining after industrial L‐tryptophan production. The mixture of L‐AAs and SCP was used as test ingredients to replace starch in N‐free diet (Table 1), and N‐free diet was used to determine protein and amino acid endogenous loss. Titanium dioxide (TiO_2_) was included in all diets at 0.5% as an indigestibility marker, and minerals and vitamins were included to meet or exceed requirements for growing pigs (Rostagno et al. 2017).

**TABLE 1:** Ingredient composition of experimental diets and analyzed (as‐fed basis).

Item (%)	L‐AAs^a^	SCP^b^	N‐free
Ingredient composition (formulated)			
Cornstarch	51.31	56.33	67.33
L‐AAs	15.02	—	—
SCP	—	10.00	—
Sucrose	20.00	20.00	20.00
Soybean oil	5.00	5.00	4.00
Dicalcium phosphate	1.96	1.96	1.96
Limestone	0.76	0.76	0.76
Sodium chloride	0.4	0.4	0.4
Vitamin supplement^c^	0.3	0.3	0.3
Mineral supplement^d^	0.25	0.25	0.25
Potassium carbonate	0.4	0.4	0.4
Antioxidant	0.01	0.01	0.01
Magnesium oxide	0.10	0.10	0.10
Cellulose	4.00	4.00	4.00
TiO_2_	0.50	0.50	0.50
Total	100	100	100
Analyzed composition			
Dry matter	91.5	91.5	91.5
Indispensable AA			
Arginine	0.40	0.56	ND
Histidine	0.42	0.21	ND
Isoleucine	0.60	0.4	ND
Leucine	0.90	0.75	0.01
Lysine	0.73	0.54	ND
Methionine	0.64	0.16	ND
Phenylalanine	0.43	0.39	ND
Threonine	0.40	0.46	ND
Tryptophan	0.09	0.41	0.04
Valine	0.59	0.52	0.01
Dispensable AA			
Alanine	ND	0.94	0.01
Aspartic acid	ND	1.01	0.04
Cysteine	ND	0.04	ND
Glutamic acid	7.42	1.31	0.03
Glycine	1.05	0.49	ND
Proline	ND	0.33	0.01
Serine	ND	0.39	ND
Tyrosine	ND	0.22	ND

Abbreviation: ND, not detected.

^a^ L‐AAs mixture included a total of 15.02% on an as‐fed basis. Its composition consisted of 0.44% L‐arginine, 8.04% L‐glutamic acid, 0.90% L‐glycine, 0.38% L‐histidine, 0.59% L‐isoleucine, 0.90% L‐leucine, 1.33% L‐lysine·HCl, 0.64% L‐methionine, 0.45% L‐phenylalanine, 0.59% L‐threonine, 0.14% L‐tryptophan, and 0.62% L‐valine.

^b^ SCP is a single cell protein biomass coproduct with analyzed amino acid concentration as fed: 5.6% arginine, 2.1% histidine, 4.0% isoleucine, 7.5% leucine, 5.4% lysine, 1.6% methionine, 3.9% phenylalanine, 4.6% threonine, 4.1% tryptophan, 5.2% valine, 9.4% alanine, 10.1% aspartic acid, 0.4% cysteine, 13.1% glutamic acid, 4.9% glycine, 3.3% proline, 3.9% serine, and 2.2% tyrosine.

^c^ Per kg of complete diet: Se, 75.00 mg/kg; vitamin A, 2000.00 IU/kg; vitamin D3, 375,000.00 IU/kg; vitamin E, 6250.00 IU/kg; vitamin K3, 750.00 mg/kg; thiamine, 500.00 mg/kg; riboflavin, 1500.00 mg/kg; pyridoxine, 500.00 mg/kg; vitamin B12, 7500.00 mcg/kg; folic acid, 250.00 mg/kg; D‐calcium pantothenate, 5000.00 mg/kg; niacin, 8750.00 mg/kg; and biotin, 37.50 mg/kg.

^d^ Per kg of complete diet: Fe, 15.00 g/kg iron sulfate; Cu, 40.00 g/kg copper sulfate; Mn, 13.00 g/kg manganese monoxide; Zn, 25.00 g/kg zinc sulfate; and I, 350.00 mg/kg calcium iodate.

### 2.3 Experimental Procedure

The animals were fed twice a day (at 07:00 and 17:00 h) based on their metabolic weight (body weight 0.75 kg) three times the estimated maintenance, with the diet mixed with water in a 1:1 ratio to avoid waste and facilitate ingestion. Throughout the experiment, the animals had free access to water. Lighting conditions were controlled, with lights turned off during the nighttime period. Ambient temperature was continuously monitored throughout the experiment and maintained within appropriate conditions for growing pigs. When necessary, 250‐W infrared heat lamps were used to maintain the environmental temperature close to the thermoneutral zone limits.

The first 5 days of each period were considered an adaptation period to the diet. On Days 6 and 7 of each period, ileal digesta samples were collected over a 10‐h period, starting immediately after the morning feeding at 07:00 h.

The ileal digesta was collected using plastic bags fixed directly to the T cannula (5 × 20 cm), which were removed after filling, replaced by new plastic bags, and immediately stored in the freezer to avoid bacterial degradation of the AA in the digesta.

After the collection period, the animals rested for 5 days, receiving a diet based on corn and soybean meals to meet the requirements according with Rostagno et al. (2017). Water was provided ad libitum throughout the study.

During the second collection period, an animal submitted to N‐free treatment presented rectal prolapse and was removed from the evaluation by a specialist.

### 2.4 Processing of Collected Material and Analysis

At the end of the experiment, ileal digesta samples were thawed, mixed, homogenized, and lyophilized per experimental unit and period. A subsample was collected for chemical analysis, totaling six replicates per treatment.

The samples of the diets and ileal digesta were analyzed to determine the content of dry matter (DM), N, TiO_2_, and AA.

#### 2.4.1 Dry Matter and Crude Protein

Dry matter (DM) was determined by oven‐drying at 105°C until constant weight according to AOAC Method 934.01 (AOAC 2019), and crude protein content was calculated from total nitrogen (N × 6.25) determined by the Kjeldahl method following AOAC Method 984.13 (AOAC 2019).

#### 2.4.2 Titanium Dioxide

Titanium dioxide (TiO_2_) concentration was determined by acid digestion followed by spectrophotometric analysis according to Myers et al. (2004) and Fowler et al. (2022), with modifications adapted to the protocol validated at the Laboratory of Animal Nutrition, Universidade Federal de Viçosa.

Feed samples and digesta were air‐ and freeze‐dried, respectively, and finely ground. Samples were accurately weighed (0.8 g for feed samples or 0.2 g for digesta samples) and transferred to macro Kjeldahl digestion tubes. Sample amounts were adjusted to ensure absorbance values fell near the midpoint of the calibration curve.

To each sample, 5 g of digester mixture (catalytic mixture) and 20 mL of concentrated sulfuric acid (95%–98% H_2_SO_4_) were added. Tubes were placed in a digestion block under a fume hood and heated slowly to 400°C. Digestion continued until the solution became greenish and translucent, indicating complete oxidation of organic matter.

After digestion, tubes were removed from the block and allowed to cool in the fume hood. Once cooled, 10 mL of hydrogen peroxide (30% P.A. grade) was added slowly to oxidize titanium species and form a stable yellow‐orange peroxo‐titanium complex. The solution was allowed to cool again after peroxide addition.

The digested material was quantitatively transferred to volumetric flasks (50 or 100 mL, depending on expected TiO_2_ concentration) using quantitative, ash‐free, rapid filtration paper. All samples and standards within the same analytical batch were diluted to the same final volume. Flasks were filled to volume with distilled water, capped, and homogenized thoroughly. Aliquots were transferred to clean polyethylene containers, and solutions were stored at 4°C until analysis.

TiO_2_ concentration was determined by measuring absorbance at 410 nm using a spectrophotometer. Immediately before reading, three drops of 30% hydrogen peroxide were added to each solution and mixed. The spectrophotometer was calibrated to zero absorbance using a blank standard (0 mg TiO_2_), followed by measurement of standard solutions and samples.

A multipoint calibration curve was prepared using TiO_2_ standards (0‐, 2.0‐, 4.0‐, 6.0‐, 8.0‐, and 10.0‐mg TiO_2_) processed through identical digestion procedures with the same amounts of digester mixture (5 g) and sulfuric acid (20 mL). The TiO_2_ used for standards was from the same batch as the indigestible marker added to feed. All glassware used for mineral analysis was prewashed with hydrochloric acid solution (200 mL/L) and rinsed thoroughly with distilled water to prevent contamination.

#### 2.4.3 Amino Acids

Total amino acid content was determined by high‐performance liquid chromatography (HPLC, CBM 20A system, Shimadzu, São Paulo, Brazil) at CBO Laboratory (Valinhos, SP, Brazil) using precolumn phenylisothiocyanate (PITC) derivatization following the validated methods of White et al. (1986) and Hagen et al. (1989).

Feed samples (50–100 mg) were subjected to acid hydrolysis in 6 mol L^−1^ HCl at 110°C for 24 h in sealed borosilicate glass tubes after oxygen removal by vacuum pump. Hydrolysates were freeze‐dried to remove acid.

Tryptophan was analyzed separately following alkaline hydrolysis with 4.2 mol L^−1^ NaOH at 110°C for 16–20 h under nitrogen atmosphere. After cooling and neutralization, tryptophan was quantified spectrophotometrically at 500 nm following Lucas and Sotelo (1980), validated according to internal procedure MA‐010.

Amino acids in dried acid hydrolysates were derivatized with phenylisothiocyanate to form phenylthiocarbamyl (PTC) derivatives detectable by UV absorbance. The three‐step derivatization procedure followed White et al. (1986) and Hagen et al. (1989) with reagent compositions specified in validated procedure MA‐009: Step 1—initial drying: Sample aliquot (40 μL) was transferred to glass tubes and freeze‐dried (~70 min); Step 2—redrying: Redrying solution (20 μL; 0.2‐N sodium acetate trihydrate:methanol:triethylamine 40:40:20 v/v/v) was added, vortexed 30 s, and freeze‐dried (~70 min), yielding white nonoily crystals; and Step 3—coupling reaction: Fresh derivatization solution with PITC (20 μL; methanol:water:triethylamine: PITC 63:9:9:9 v/v/v/v) was added, vortexed 30 s, and allowed to react 20 min at room temperature before final freeze‐drying. Dried PTC‐amino acids were reconstituted in diluent buffer and filtered (0.45 μm) for HPLC analysis.

Calibration accuracy for amino acid standards (certified reference materials in 0.1 mol L^−1^ HCl) was subjected to identical derivatization procedures as samples, including the same volumes, timing, and drying steps. This parallel treatment ensures accurate calibration since PTC derivatization yield depends on reagent quality, reaction time, and temperature.

PTC‐amino acids were separated on a C18 reverse‐phase column at 38°C using gradient elution with Mobile Phase A (0.14 mol L^−1^ sodium acetate buffer pH 6.4 with 0.05% triethylamine) and Mobile Phase B (acetonitrile: water 60:40 v/v) at 1.0 mL min^−1^ flow rate. Detection was at 254 nm using a photodiode array detector (SPD‐M20A). Peak identification was based on retention time matching with derivatized standards, and quantification used external calibration by peak area integration. Complete gradient program and column specifications are detailed in validated procedure MA‐009.

Multipoint calibration curves were constructed for each amino acid using freshly derivatized standards (*r* ^2^ ≥ 0.999 required) for quality control. Each analytical batch included (1) reagent blanks to monitor contamination (acceptance: signal < 30% of first calibration point); (2) certified reference materials (recovery 80–110%); and (3) duplicate independent sample analyses (CV ≤ 10%). Analytical performance was continuously monitored using control charts with statistical rules (7‐point trend and 10‐point shift detection) to identify instrumental drift or systematic problems. The laboratory participates in proficiency testing schemes with satisfactory results.

All analyses were performed in duplicate, and results are reported as mean ± standard deviation (g/100 g protein or mg/g sample as appropriate).

### 2.5 Calculation of Standardized Digestible

The SID for CP and AA of the industrial L‐AAs and SCP were calculated using a direct method. The values calculated for this diet represented the values of the test ingredients. The following formulas were used to determine the SID (Sakomura and Rostagno 2016) and Ileal basal endogenous losses (BEL):

$$\mathrm{FI}1\;\left(\text{indigestibility marker of the test diet}\right)=\frac{\%\mathrm{TiO}2\;\text{test diet}}{\%\mathrm{TiO}2\;\text{test digest}}$$

$$\text{Apparent ileal digestibility}\;\left(\mathrm{AID}\right)\;\text{of}\;\mathrm{CP}\left(\%\right)=\frac{\left[\left(\%\mathrm{CP}\;\text{diet}-\left(\%\mathrm{CP}\;\text{digest}\times \mathrm{FI}1\right)\right)\right]}{\%\mathrm{CP}\;\text{diet}}\times 100$$

$$\mathrm{SID}\;\mathrm{CP}\;\left(\%\right)=\frac{\left[\left(\%\mathrm{CP}\;\text{diet}-\left(\%\mathrm{CP}\;\text{digest}\times \mathrm{FI}1\right)-\left(\%\mathrm{CP}\;\text{diet}\times \mathrm{FI}2\right)\right)\right]}{\%\mathrm{CP}\;\text{diet}}\times 100$$

FI2 = indigestibility marker of the N‐free diet.

$$\mathrm{AID}\;\mathrm{AA}\;\left(\%\right)=\frac{\left[\left(\mathrm{mg}\;\mathrm{AA}/g\;\text{diet}-\left(\mathrm{mg}\;\mathrm{AA}/g\;E1\times \mathrm{FI}1\right)\right)\right]}{\mathrm{mg}\;\mathrm{AA}\;\text{in diet}}\times 100$$

E1 = digesta test diet

$$\mathrm{SID}\;\mathrm{AA}\;\left(\%\right)=\frac{\left[\left(\mathrm{mg}\;\mathrm{AA}/g\;\text{diet}-\left(\mathrm{mg}\;\mathrm{AA}/g\;E1\times \mathrm{FI}1\right)-\left(\mathrm{mg}\;\mathrm{AA}/g\;E2\times \mathrm{FI}2\right)\right)\right]}{\mathrm{mg}\;\mathrm{AA}\;\text{in diet}}\times 100$$

E2 = digesta N‐free diet.

The following formula was used to determine basal ileal endogenous amino acid losses (Adeola et al. 2016).

BELofAA:

$$\mathrm{BEL}\;\text{of}\;\mathrm{AA}\;\left(\%\right)=\mathrm{mg}\;\mathrm{AA}/g\;\text{digest}\times \left(\%\mathrm{TiO}2\;N-\text{free diet}/\%\mathrm{TiO}2\;\text{digest}\right)$$

### 2.6 Statistical Analysis

The digestibility was established from the mean values obtained from the replicates, and later, the standard deviations of the averages were calculated, and the replicates with values higher than 1.5 of the standard deviation were considered outliers. Analysis of variance was performed to evaluate differences between the means of the treatments; the differences were considered significant if *p* < 0.05. The analyses were performed using R Version 4.3.1 software (RStudio, 2023).

## 3 Results

The coefficients of AID and SID are presented in Table 2, whereas the detailed digestibility values are shown in Table S1. The CP for the sources of AA for pigs (L‐AAs and SCP) exhibits significant differences (*p* < 0.05) showing that L‐AAs obtained higher CP digestibility (101.3%) compared to SCP (73.4%).

**TABLE 2:** Apparent ileal digestible and standardized ileal digestible coefficients of CP and AAs of L‐AAs and a SCP biomass coproduct determined by growing pig ileal digestibility assay.

Item	Apparent ileal digestibility			Standardized ileal digestibility			
	L‐AAs	SCP	*p* value	L‐AAs	SCP	*p* value	SEM
Crude protein (%)	90.1	63.9	< 0.001	101.3	73.4	< 0.001	2.4
Indispensable AA							
Arginine (%)	91.9	73.7	0.002	101.4	80.4	0.001	2.8
Histidine (%)	96	67.3	< 0.001	99.8	74.9	< 0.001	2.2
Isoleucine (%)	96.7	61.4	< 0.001	101	67.9	< 0.001	2.4
Leucine (%)	95.5	61.5	< 0.001	100.7	67.7	< 0.001	2.5
Lysine (%)	97.7	65.5	< 0.001	100.3	69.1	< 0.001	1.7
Methionine (%)	98.9	59.5	< 0.001	100.2	65	< 0.001	2.3
Phenylalanine (%)	95	59.8	< 0.001	101	66.3	< 0.001	2.1
Threonine (%)	88.3	60	< 0.001	100.6	70.8	< 0.001	2.7
Tryptophan (%)	74.9	81.8	0.078	108.4	89.3	0.001	2.4
Valine (%)	95.1	62.7	< 0.001	101.5	70.1	< 0.001	2.4
Dispensable AA							
Alanine (%)	ND^1^	56	—	ND	61.5	—	—
Aspartic acid (%)	ND	71	—	ND	74.3	—	—
Glutamic acid (%)	98.5	59.7	< 0.001	99.4	64.6	< 0.001	2.4
Glycine (%)	89.6	56.7	0.017	100.7	80.3	0.1	7.8
Serine (%)	ND	58.2	—	ND	71.1	—	—
Tyrosine (%)	ND	54.9	—	ND	63.7	—	—

*Note:* The tested model is significant *p* < 0.05. The values were represented by six observations. Baseline endogenous losses were determined by the N‐free diet (g/kg of daily DM intake): CP, 13.88 lysine, 0.21; methionine, 0.09; threonine, 0.54; tryptophan, 0.33; Arginine, 0.41; valine, 0.42; isoleucine, 0.29; leucine, 0.51; histidine, 0.17; phenylalanine, 0.28; alanine, 0.57; cysteine, 0.05; tyrosine, 0.21; glycine, 1.27; serine, 0.55; proline, 3.19; glutamic acid, 0.69; aspartic acid, 0.37.

Abbreviations: L‐AAs, L‐amino acids mixture; ND, not detected; SCP, single‐cell protein biomass coproduct; SEM, standard error of the mean.

Regarding indispensable AA, a significant difference (*p* < 0.05) was observed in SID for L‐tryptophan, L‐phenylalanine, L‐histidine, L‐isoleucine, L‐leucine, L‐lysine, L‐methionine, L‐threonine, and L‐valine. Mixture of industrial L‐AAs also showed better L‐tryptophan digestibility (108.3%), compared to SCP (89.2%). For the other AA, all comparisons between L‐AAs and SCP showed differences (*p* < 0.05), with superior digestibility for the source L‐AAs. Dispensable AA showed a difference for all comparisons between L‐AAs and SCP, with L‐AAs presenting higher SID. In addition, glutamic acid showed differences (*p* < 0.05) between the sources analyzed, with better digestibility values for the L‐AAs source. However, for glycine, no difference was observed.

The baseline endogenous losses were determined by the N‐free diet (g/kg of daily DM intake): CP, 13.88 lysine, 0.21; methionine, 0.09; threonine, 0.54; tryptophan, 0.33; arginine, 0.41; valina, 0.42; isoleucine, 0.29; leucine, 0.51; histidine, 0.17; phenylalanine, 0.28; alanine, 0.57; cysteine, 0.05; tyrosine, 0.21; glycine, 1.27; serine, 0.55; proline, 3.19; glutamic acid, 0.69; and aspartic acid, 0.37.

## 4 Discussion

The present study evaluated the digestibility of L‐AAs and SCP biomass coproduct for growing pigs. As expected, the diet containing L‐AAs mixture showed higher digestibility values for CP and AA, confirming the premise that industrial AA, due to its purified and highly bioavailable form, is more easily absorbed (Miranda et al. 2015). The SIDs of CP were 101.3% for L‐AAs and 73.4% for SCP. The baseline endogenous losses determined in this study were consistent with the values recently reported by Park et al. (2024), indicating that the correction applied for endogenous amino acid losses was accurate and appropriate for estimating SID values. Although the SCP obtained low SID for CP than L‐AAs, it presents a significant CP content, indicating that, even after the extraction of specific AA, the residual biomass still contains considerable amounts of AA, resulting in a significant CP digestibility.

In previous studies, Espinosa et al. (2021), Almeida et al. (2014), and Sulabo et al. (2013) found SID for CP of 86.3%, 91.3%, and 91.8%, respectively, for different sources of AA biomass. These values are higher than those observed in the present study, possibly due to the production techniques of industrial AA, which often include the use of enzymes to accelerate the process and increase the nutritional value of the biomass. However, these same processes can also raise the cost of the final product.

When compared with conventional protein sources, Kong et al. (2014) reported a SID of CP of 88.8% for soybean meal, which is higher than the value observed for SCP (73.40%) in the present study. In contrast, the same study reported a SID of CP of 63.5% for meat meal, which is lower than that observed for SCP, indicating that SCP presents an intermediate digestibility profile among conventional protein sources.

However, SCP and soybean meal present distinct antinutritional profiles. Although soybean meal contains trypsin inhibitors and allergenic storage proteins that can be reduced through fermentation (Nadar et al. 2024; van der Spiegel et al. 2020), SCP is primarily characterized by high nucleic acid content (0%–15% of dry matter) and potential novel allergenic proteins (Najafpour 2015). Although SCP lacks the classical plant antinutrients found in soybean meal, its use in animal nutrition requires careful management of nucleic acid levels and consideration of potential immunological effects reported in some bacterial SCP products (Najafpour 2015). Therefore, SCP represents a sustainable alternative protein source with intermediate digestibility, though direct quantitative comparisons of specific antinutritional factors between SCP and conventional protein sources remain limited in the literature.

In addition, ingredients such as fish meal, widely used to improve the protein quality of diets and which have approximately 87% of SID of CP (Rostagno et al. 2024), are expensive. In this way, SCP emerges as an economical and viable alternative.

Regarding the digestibility of AA, SCP biomass stands out for the SID of 89.25% for L‐tryptophan, a value that corroborates the findings of (Espinosa et al. 2021), who reported a high concentration and SID of 95.3% for L‐tryptophan in biomass coproducts after L‐tryptophan extraction. Similar results were observed by Almeida et al. (2014) for L‐threonine biomass and by Sulabo et al. (2013) for L‐lysine biomass. These findings suggest that the extraction of specific AA from the fermented biomass is not 100% efficient, resulting in a retained biomass with high concentrations of fermented AA, in addition to other AA.

For conventional protein sources, Kong et al. (2014) reported SID values for L‐tryptophan of 92.3% for soybean meal and 78.5% for meat meal, whereas the SCP evaluated in the present study showed a SID of 81.8% for this AA, again demonstrating a relevant digestibility value that supports its potential to partially replace conventional protein ingredients.

The values of SID of industrial AA obtained in this study differed from those reported by Chung and Baker (1992). In the first case, the CP value was 96.0%, whereas the present study showed 101.3%. Additionally, the digestibility coefficients were higher for all essential AA, including L‐arginine (101.4% vs. 100.5%), L‐phenylalanine (101.0% vs. 99.4%), L‐isoleucine (101.0% vs. 100.1%), L‐leucine (100.7% vs. 97.2%), L‐lysine (100.3% vs. 99.3%), L‐methionine (100.2% vs. 99.2%), L‐threonine (100.6% vs. 98.2%), L‐tryptophan (108.3% vs. 97.9%), and L‐valine (101.5% vs. 98.5%), with similar values only for L‐histidine (99.8% vs. 99.3%).

A similar trend was observed when comparing the results with the reference values from BTPS, proposed by Rostagno et al. (2024), which is currently one of the main sources for formulating diets for poultry and pigs. When compared to our findings, the values reported by BTPS showed lower digestibility for several AA, such as arginine (95.5%), phenylalanine (95.2%), glycine (97.0%), glutamic acid (99.2%), isoleucine (97.1%), leucine (95.4%), lysine (98.1%), methionine (99.5%), threonine (96.8%), tryptophan (99.0%), and valine (95.5%). In our study, all these AA showed higher digestibility, except for histidine, which showed a slight reduction (99.79% vs. 100.0%).

These differences may be attributed to the genetic evolution of the animals, the improvement of industrial processes in AA production, and the advancement of laboratory analysis techniques. These factors reinforce the need for updating the ileal digestibility currently used in diet formulation for pigs. Therefore, our findings highlight the need for revising the SID values for industrial AA, considering that was obtained for L‐AAs were close to 100%. Moreover, our results suggest that the extraction of specific AA from the fermented biomass is not 100% efficient, so SCP has good values of SID for indispensable and dispensable AA, highlighting its viability as a source of AA for pigs.

The results of this study indicate a complete absorption of AA from industrial AA, with SID close to 100%. The SCP biomass, on the other hand, is a viable source of AA for pigs, with an average SID of 73.4% for CP and 72.1% and 69.2% for dispensable and indispensable AA, respectively. The determined SID values for L‐AAs and SCP can be used for precise swine diet formulation.

## Funding

This study was supported by INCT – CA National Institute of Science and Technology Animal Sciences, CAPES – Coordination for the Improvement of Higher Education Personnel, CNPq – National Council for Scientific and Technological Development, and FAPEMIG – Foundation for Research Support of the State of Minas Gerais.

## Conflicts of Interest

The authors declare no conflicts of interest.

## Supporting information


**Table S1:** Apparent ileal digestible (AID) and standardized ileal digestible (SID) contents of CP and AA (% of dry matter) in diets containing L‐AAs and SCP.

## Data Availability

The data that support the findings of this study are available from the corresponding author upon reasonable request.
