# Supplementary material for: Standardized Ileal Digestibility of Amino Acids in L‐Amino Acids Sources and Single Cell Protein Biomass Coproduct for Pigs
Source: Anim Sci J. 2026 Apr 4;97(1):e70177. doi: 10.1111/asj.70177 (PMC13049558; doi:10.1111/asj.70177)
Supplement: Supplementary file 1 — Table S1: Apparent ileal digestible (AID) and standardized ileal digestible (SID) contents of CP and AA (% of dry matter) in diets containing L‐AAs and SCP. [file ASJ-97-e70177-s001.docx]

**Supporting Information:** Table S1: Apparent ileal digestible values (AID) and standardized ileal digestible values (SID) of CP and AA of the L-AAs and SCP diets (Dry Matter).

**Table S1.** Apparent ileal digestible (AID) and standardized ileal digestible (SID) contents of CP and AA (% of dry matter) in diets containing L-AAs and SCP.

|  | **L-AAs^a^** | | **SCP^b^** | |
| --- | --- | --- | --- | --- |
| **Item** | **AID** | **SID** | **AID** | **SID** |
| Crude Protein, % | 68.944 | 77.533 | 44.391 | 51.012 |
| ***Indispensable AA*** |  |  |  |  |
| Arginine, % | 1.222 | 1.349 | 2.749 | 2.999 |
| Histidine, % | 1.210 | 1.257 | 0.875 | 0.974 |
| Isoleucine, % | 3.152 | 3.293 | 1.621 | 1.793 |
| Leucine, % | 4.890 | 5.156 | 2.905 | 3.198 |
| Lysine, % | 3.183 | 3.270 | 2.312 | 2.438 |
| Methionine, % | 3.549 | 3.598 | 0.666 | 0.728 |
| Phenylalanine, % | 2.841 | 3.019 | 1.547 | 1.716 |
| Treonine, % | 2.579 | 2.936 | 1.818 | 2.144 |
| Tryptophan, % | 0.494 | 0.715 | 1.726 | 1.883 |
| Valine, % | 2.966 | 3.166 | 2.108 | 2.354 |
| ***Dispensable AA*** |  |  |  |  |
| Alanine, % | ND^c^ | ND | 3.292 | 3.617 |
| Aspartic Acid, % | ND | ND | 4.465 | 4.674 |
| Glutamic Acid, % | 48.642 | 49.105 | 4.794 | 5.185 |
| Glycine, % | 3.460 | 3.887 | 1.837 | 2.601 |
| Serine, % | ND | ND | 1.368 | 1.672 |
| Tyrosine, % | ND | ND | 0.982 | 1.140 |

^a^L-AAs: L-amino acids mixture

^b^SCP: single cell protein biomass co-product.

^c^ND = Not detected.
